# Salvage Latarjet may provide worse outcomes in terms of recurrent instability and returning to sports compared to primary Latarjet: a systematic review of comparative studies

**DOI:** 10.1186/s12891-024-07593-w

**Published:** 2024-06-27

**Authors:** Chunsen Zhang, Songyun Yang, Long Pang, Tao Li, Yinghao Li, Haoyuan Wang, Yizhou Huang, Xin Tang

**Affiliations:** 1 Sports Medicine Center, West China Hospital, Sichuan University, Chengdu, 610041 China; 2 Department of Orthopedics and Orthopedic Research Institute, West China Hospital, Sichuan University, Chengdu, 610041 China; 3 Santai County People’s Hospital, Mianyang, 621199 China; 4 Department of Orthopedic Surgery and Orthopedic Research Institute, Laboratory of Stem Cell and Tissue Engineering, State Key Laboratory of Biotherapy, West China Hospital, Sichuan University, Chengdu, 610041 China; 5 Department of Orthopedics, Shanghai Sixth People’s Hospital Affiliated to Shanghai Jiao Tong University School of Medicine, Shanghai, 200233 China

**Keywords:** Shoulder instability, Latarjet procedure, Primary Latarjet, Salvage Latarjet, Outcome

## Abstract

**Background:**

The Latarjet procedure (LP) is performed as a primary stabilization procedure (primary LP) and a salvage procedure when an earlier shoulder stabilization procedure has failed (salvage LP). However, whether primary LP or salvage LP provides better outcomes for anterior shoulder instability remains unknown.

**Methods:**

Two independent reviewers performed the literature search based on the PRISMA guidelines. A comprehensive search of PubMed, Embase, web of science and Cochrane Library was performed from their inception date to December 4, 2023. Inclusion criteria mainly included the comparison of postoperative outcomes between primary and salvage LP, English language, and full text availability. Two reviewers independently examined the literature, collected data, and evaluated the methodological robustness of the included studies. The Methodological Index for Nonrandomized Studies was used to evaluate the quality of nonrandomized studies. Recurrent instability, complications, reoperations, return to sports, patient-reported outcomes, and range of motion were assessed. Statistical evaluations were conducted using Manager V.5.4.1 (The Cochrane Collaboration, Software Update, Oxford, UK).

**Results:**

Twelve studies were included in the systematic review, with 940 shoulders undergoing primary LP and 631 shoulders undergoing salvage LP. Statistically significant differences in favor of primary LP were found in 2 of the 11 and 2 of 4 included studies in terms of recurrent instability and returning to the same sports (RTS) at preinjury level, respectively. In terms of the visual analog scale, subjective shoulder value and the Western Ontario Shoulder Instability Index, 2 of the 4, 1 of the 3 and 1 of the 3 included studies reported statistically significant differences in favor of primary LP. Differences were not noticed regarding complications, reoperations, the time to RTS, the Rowe score, the Athletic Shoulder Outcome Scoring System, and forward flexion.

**Conclusion:**

Current evidence suggests that compared with primary LP, salvage LP may provide inferior postoperative outcomes in terms of recurrent instability and the rate of RTS at preinjury level. Primary and salvage LP may yield comparable efficacy in terms of complications, reoperations, the rate of RTS, the time to RTS, pain, shoulder function, and range of motion.

**Prospero id:**

CRD42023492027.

**Supplementary Information:**

The online version contains supplementary material available at 10.1186/s12891-024-07593-w.

## Introduction

The shoulder is the most commonly dislocated joint, with anterior instability being the predominant form of shoulder instability [1]. The incidence of anterior shoulder instability is estimated to range from 1 to 2% in the general population, while it tends to be significantly higher among young and physically active individuals [2–5]. Currently, there exist numerous surgical procedures aimed at restoring shoulder stability, among which the Latarjet procedure (LP) stands out as one of the most frequently employed techniques for addressing anterior shoulder instability [6, 7].

The LP, initially described by Dr. Michel Latarjet in 1954, has gained widespread recognition for its efficacy. However, there have been divergent opinions regarding the application of LP over the years. Some shoulder surgeons advocate for LP as a primary intervention for anterior shoulder instability due to its low recurrence rate and high rate of return to sport [8–14]. Conversely, others caution against using LP as a primary procedure due to its nonanatomic nature and associated complications [15–17]. Previous studies have reported that LP can achieve good results as both a primary and salvage procedure in managing anterior shoulder instability [18–20], but recent research suggests that salvage LP may carry higher risks of re-dislocation and inferior clinical outcomes compared to primary LP [21, 22]. Which is better in terms of clinical efficacy between primary and salvage LP remains unknown, prompting increased attention from surgeons on this matter. Clarifying this issue will enhance our understanding of both primary and salvage LP procedures while potentially influencing their indications.

The purpose of this systematic review was to compare the postoperative outcomes between primary LP and salvage LP. Given that salvage LP is a revision procedure for previous failed shoulder surgeries, we hypothesized that patients who underwent salvage LP would have inferior postoperative outcomes compared with those who underwent primary LP.

## Methods

### Search strategy

This study has been registered on PROSPERO (ID CRD42023492027). This systematic review was performed according to PRISMA (Preferred Reporting Items for Systematic Reviews and Meta-Analyses) guidelines [23]. Two independent reviewers performed an electronic search in 4 databases (PubMed, Embase, web of science and Cochrane Library) from their inception date to December 4, 2023. The following search items were used: (shoulder instability OR recurrent shoulder instability OR recurrent shoulder anterior instability OR shoulder dislocation OR shoulder anterior dislocation OR recurrent shoulder anterior dislocation) AND (Latarjet OR Bristow-Latarjet OR coracoid transfer) AND (primary OR first-line OR salvage OR revision OR reoperation). The reference lists of previous relevant studies were also reviewed.

### Eligibility criteria

The inclusion criteria were: (1) Clinical studies comparing postoperative outcomes of primary LP and salvage LP; (2) Studies published in the English language; (3) Studies for which the full text was accessible. The exclusion criteria were: (1) Nonclinical studies (e.g., in vitro experiments, animal studies, or cadaveric studies); (2) Other types of clinical studies (e.g., case reports, commentaries, editorials, etc.) (3) Patients enrolled with concomitant shoulder diseases such as rotator cuff injuries, severe osteoarthritis, infections, and tumors.

### Study selection

Two reviewers (C.Z. and S.Y.) independently assessed the titles and abstracts of the retrieved literature, resulting in exclusion of most articles at this stage. The remaining articles were further evaluated for eligibility by reviewing their full text. Any discrepancies during the screening process were resolved through discussion between the abovementioned researchers and a senior author (X.T.).

### Data extraction

The data of interest included study characteristics (author, year of publication, study design, level of evidence, surgical details, mean follow-up duration and sample size), patient demographic data (proportion of male patients and mean age), injury characteristics (glenohumeral joint bone loss), and postoperative clinical outcomes (recurrent instability, complications, reoperations, return to sports, patient-reported outcomes, and range of motion).

Recurrent instability was defined as the occurrence of postoperative redislocation or subluxation. Complications were defined as adverse events related to the LP (hematoma, infection, nerve palsy, etc.). The patient-reported outcomes included the subjective shoulder value (SSV) [22, 24, 25], Western Ontario Shoulder Instability Index (WOSI) [22, 24, 26], Athletic Shoulder Outcome Scoring System (ASOSS) [24, 27, 28], Rowe score [25, 27, 28] and visual analog scale (VAS) [25, 27–29].

### Quality assessment

Two independent reviewers (C.Z. and S.Y.) rigorously evaluated the methodological quality of the included studies using Methodological Index for Nonrandomized Studies (MINORS) [30], and any disagreements during this process were resolved by the senior author (X.T.). The MINORS is a validated scoring tool for nonrandomized studies, which consisted of 12 items (4 for comparative studies and 8 for noncomparative studies). Each item was suggested scored as 0 (not reported), 1 (reported but inadequate) and 2 (reported and adequate). The maximum scores for noncomparative studies and comparative studies are 16 points and 24 points, respectively. In this study, the MINORS score for nonrandomized studies was graded as follows: 0 to 5, very low quality; 6 to 10, low quality; 11 to 15, fair quality and more than 16, good quality [31].

### Statistical analysis

Pooling of results should be avoided in systematic reviews that include low-quality studies (LOE III - IV), rendering meta-analysis inappropriate for this study. Statistical analyses were conducted using Manager V.5.4.1 (The Cochrane Collaboration, Software Update, Oxford, UK). The differences between primary LP and salvage LP were determined using forest plots. Using I^2^ statistics, we assessed and defined the heterogeneity of each qualified study. I^2^ values of 25%, 50%, and 75% were considered to indicate low, medium, and high heterogeneity, respectively. If the I^2^ value exceeded 50%, a sensitivity analysis was performed to investigate the source of heterogeneity, aiming to further mitigate the impact of studies with substantial heterogeneity on the conclusions. Statistical significance was defined as a *P* value < 0.05.

The minimal clinically important difference (MCID) was used to determine the clinical significance of a change in scores on an outcome measure. The MCID values for VAS score and WOSI score after LP were 1.7 and 254.9 [32]. Since the MCID values for SSV after LP was not reported in literature, the value for SSV score after massive rotator cuff repair was adopted in this study, which was 13.7 [33].

## Results

### Study selection

**Fig. 1:**
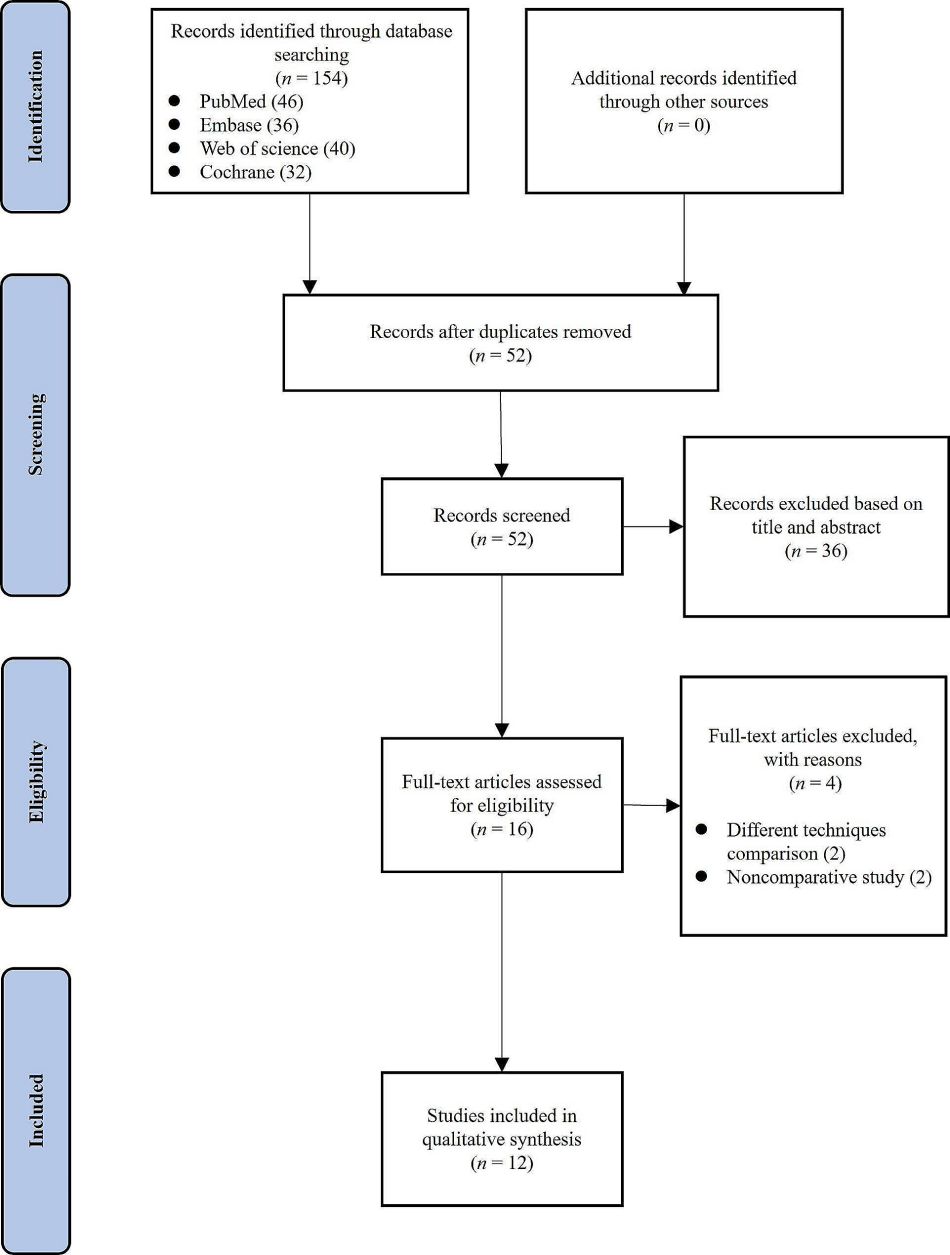
Flowchart diagram of the study selection

A total of 154 articles were retrieved through a comprehensive literature search, from which 102 duplicate articles were excluded. According to the inclusion and exclusion criteria, 12 studies were included in this systematic review. The reasons for exclusion at each step of the screening process were shown in Fig. 1.

### Quality assessment

The mean MINORS score of the twelve studies was 20.8 (SD, 1.5; range, 18–23), with all studies demonstrating good methodological quality [21, 22, 24–29, 34–37] (Table 1).

**Table 1:** The characteristics and MINORS scores of the included studies

First Author, Year	Study Design, LOE	Sample Size - Shoulders		Sex, M/F, *n*		Mean Age, Mean ± SD, y		Follow - up, Mean ± SD, m		MINORs score
		Primary	Salvage	Primary	Salvage	Primary	Salvage	Primary	Salvage	
Ranalletta, 2018 [27]	RCS, 3	18	31	48/0		22.8 ± 4.5		48 ± 21		20
Rossi, 2018 [28]	RCS, 3	46	54	40/6	52/2	25.7 ± 7.3	27.3 ± 8.3	58 ± 21		22
Flinkkilä, 2019 [22]	RCS, 3	47	52	36/11	42/10	32 ± 14	33 ± 8	34.8 ± 16.8 *	55.2 ± 31.2 *	21
Buckup, 2020 [24]	RCS, 3	38	9	40/7		24.5 ± 5.9		27.8 ± 7.6		19
Frantz, 2020 [34]	PCS, 2	19	46	59/6		24.5 ± 8.2		6.0		22
Updegrove, 2020 [35]	RCS, 3	54	103	46/8	91/12	31.4 ± 11.1 *	27.1 ± 8.9 *	7.8 ± 11.0	7.0 ± 13.2	21
Werthel, 2020 [29]	RCS, 3	216	20	186/30	15/5	27.7 ± 9.2	28.9 ± 9.7	40.8 ± 9.6		20
Yapp, 2020 [26]	RCS, 3	145	60	133/12	54/6	NR		69.3	81.2	20
Davey, 2021 [25]	RCS, 3	150	50	150/0	50/0	22.5 ± 5.2	23.3 ± 3.2	39.8 ± 23.8		23
Rodkey, 2021 [21]	RCS, 3	99	135	96/3	130/5	25.9 ± 6.2	27.5 ± 6.5	57.6	62.4	21
Gambhir, 2022 [36]	RCS, 3	54	23	49/5	21/2	30 ± 10	26 ± 6	3.0		23
Alfaraidy, 2023 [37]	RCS, 3	54	48	83/13		26.7 ± 8.9		7.2 ± 2.8		18

LOE: level of evidence; RCS: retrospective cohort study; PCS: prospective cohort study; LP: Latarjet procedure; M/F: male/female; y: year; m: month; MINORS: Methodological Index for Nonrandomized Studies; NR: not reported; Statistically significant differences observed between the two groups are denoted by asterisks

### Description of studies

Twelve studies were included in this study, including 11 retrospective cohort studies [21, 22, 24–29, 35–37] and 1 prospective cohort study [34]. All included studies were published between 2018 and 2023. A total of 1564 patients were enrolled, 90.9% of whom were male. 940 patients underwent primary LP (primary LP group) and 631 patients underwent salvage LP (salvage LP group). The surgical approach utilized in ten of the twelve studies were open surgery, whereas the other two were arthroscopic or mini-open surgery. The characteristics and surgical details of the included studies are summarized in Tables 1 and 2, respectively.

**Table 2:** The surgical details of the included studies

Study	Surgical Approach	Indication	Coracoid Graft Fixation	Prior Procedures in the Salvage LP Group, *n*/*N*	Glenoid Bone Loss, Mean ± SD or Mean (Range), %		Hill-Sachs Lesions
					Primary	Salvage	
Ranalletta, 2018 [27]	Open LP	Competitive rugby players who had a glenoid bone defect greater than 20% shown on the preoperative computed tomography scan.	2 partially threaded cortical screws (3.5-mm diameter)	Open Bankart repair: 6/31 Arthroscopic Bankart repair: 25/31 Number of previous operations, mean (range): 1.2 (1–3)	28 (20–36)		NR
Rossi, 2018 [28]	Open LP	Competitive athletes who had a glenoid bone defect greater than 20% shown on the preoperative computed tomography scan prior to the LP.	2 partially threaded cannulated cortical screws (3.5 mm diameter)	Open Bankart repair: 10/68 Arthroscopic Bankart repair: 44/68 Revision arthroscopic Bankart repair: 14/68 Number of previous operations, mean (range):1.26 (1–3)	25(20–36)	26(20–38)	NR
Flinkkilä, 2019 [22]	Open LP	Primary LP: participation in contact or collision sports, or a glenoid or humeral bony defect that was considered a contraindication for Bankart repair. Salvage LP: a failed arthroscopic Bankart repair, regardless of the bony pathology.	Two 4.5 mm cannulated screws	Number of previous arthroscopic Bankart repairs (n/N) 1: 44/52 2: 7/52 3: 1/52	21 ± 8 *	24 ± 9 *	Off-track/on-track, n_1_/n_2_ Primary: 14/33 * Salvage: 27/25 * The width of Hill–Sachs interval, mm Primary: 13 ± 9 * Salvage: 17 ± 9 *
Buckup, 2020 [24]	Arthroscopic LP	A pre-operative unidirectional traumatic anterior shoulder instability Gerber type IIB, a high level of athletic performance, and an ISIS score of ≧ 4.	2 titanium screws	NR	NR		NR
Frantz, 2020 [34]	Open LP	NR	NR	NR	Range, %: n/total 0: 9/65 1–10: 6/65 11–20: 31/65 21–30: 19/65		NR
Updegrove, 2020 [35]	Open LP	NR	2 screws	NR	25.9 ± 6.6	23.6 ± 9.0	NR
Werthel, 2020 [29]	Mini-open/ arthroscopic LP	NR	Two 4-mm/3.5-mm cannulated cancellous screws	Arthroscopic Bankart repair: 20/20	The presence of glenoid lesion, n/N Primary: 163/216 Salvage: 12/20		The presence of Hill-Sachs lesions, n/N Primary: 163/216 * Salvage: 9/20 *
Yapp, 2020 [26]	Open LP	Primary LP: evidence of significant glenoid bone loss measuring greater than 20%; arthroscopic evidence of an engaging Hill–Sachs lesion when the arm was placed in 90° abduction and external rotation. Salvage LP: failure of a previous open or arthroscopic soft-tissue stabilization procedure for recurrent anterior traumatic instability.	Two fully threaded 3.5 mm lag screws	Arthroscopic stabilization: 45/60 Open stabilization, e.g., Bankart repair: 15/60	NR		NR
Davey, 2021 [25]	Open LP	Based on patients’ risk factors, including age, sport played and level of sport, the presence of off-track Hill-Sachs lesions, and the percentage of glenohumeral bone loss.	2 standard 3.5 mm, partially threaded cancellous screws	NR	12.2 ± 8.1 *	17.6 ± 8.5 *	Off-track/on-track, n_1_/n_2_ Primary: 68/82 Salvage: 20/30
Rodkey, 2021 [21]	Open LP	Primary LP: anterior instability and > 15–20% bone loss. Salvage LP: recurrent anterior instability after previous failed attempts, any amount of bone loss, or arthroscopic evidence of engaging Hill-Sachs lesion.	NR	Number of previous operations, mean (range): 1.4 (1–5)	NR		NR
Gambhir, 2022 [36]	Open LP	Primary LP: instability arising from critical bone loss. Salvage LP: failed arthroscopic instability repair.	2 screws	NR	17 ± 7.9	15 ± 5.9	Off-track/on-track, n_1_/n_2_ Primary: 26/28 Salvage: 9/14 The depth of Hill-Sachs lesions, mean ± SD, mm Primary: 4.8 ± 2.8 Salvage: 4.6 ± 2.6
Alfaraidy, 2023 [37]	Open LP	Primary LP: a diagnosis of recurrent anterior shoulder instability, with isolated glenoid bone loss greater than 20% on preoperative CT en face view, or combined bone defects on the glenoid and the humeral head (‘‘bipolar lesion’’). Salvage LP: failure of prior treatment with soft tissue repair procedure.	2 cannulated 4.0- or 4.5-mm partially threaded cancellous screws	NR	NR		NR

LP: Latarjet procedure; NR: not reported; n: number; N: the sample sizes of the respective groups; mm: millimeter; Statistically significant differences observed between the two groups are denoted by asterisks

### Recurrent instability

**Fig. 2:**
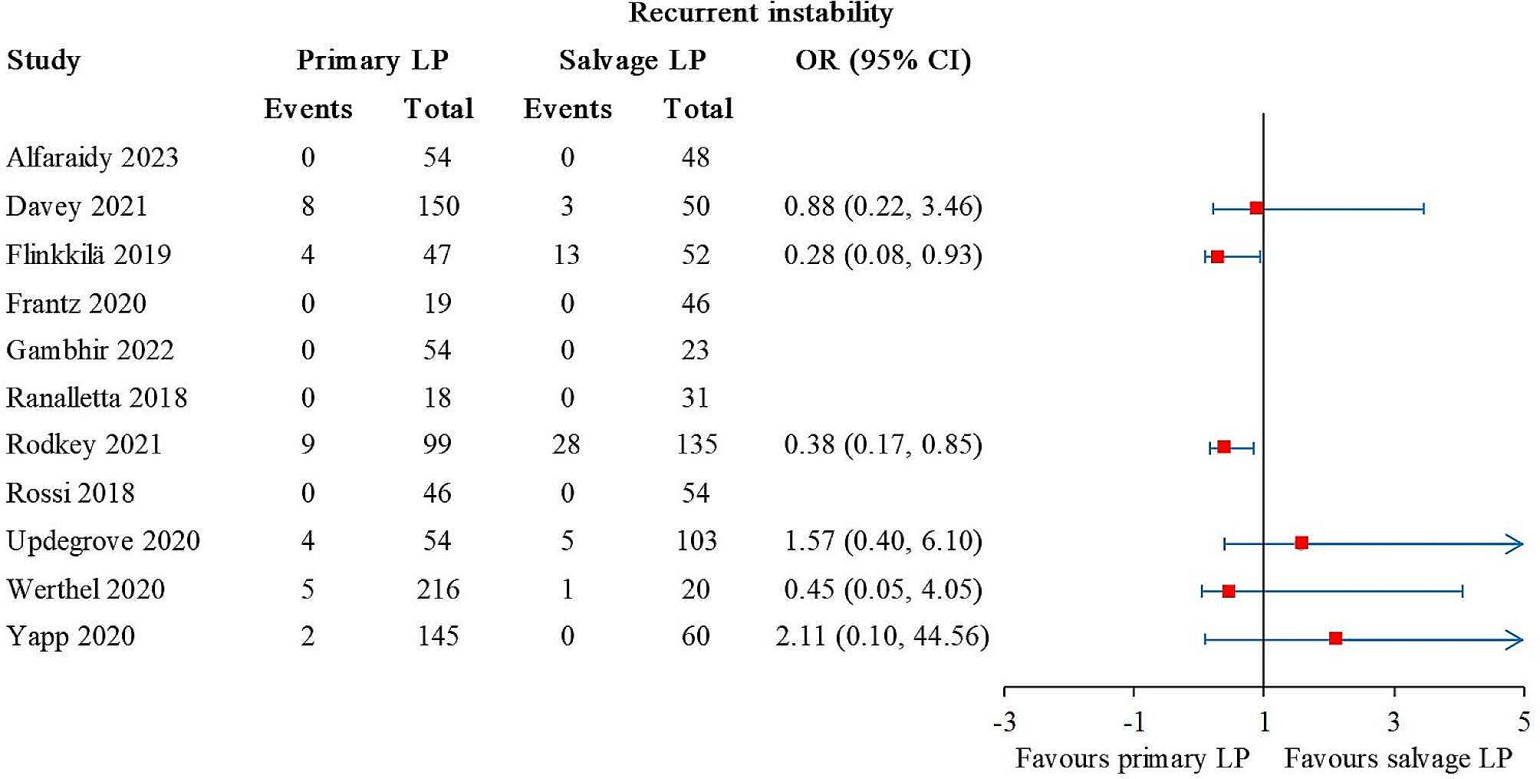
Forest plot for recurrent instability. The arrows represent values exceeding the axis scale. (CI, confidence interval; LP, Latarjet procedure.)

Recurrent instability was reported in 11 studies, comprising 902 primary LP and 622 salvage LP [21, 22, 25–29, 34–37]. Statistically significant differences were found in 2 out of 11 studies [21, 22], and the results favored primary LP (Fig. 2). The overall I^2^ value was 10%.

### Complications

**Fig. 3:**
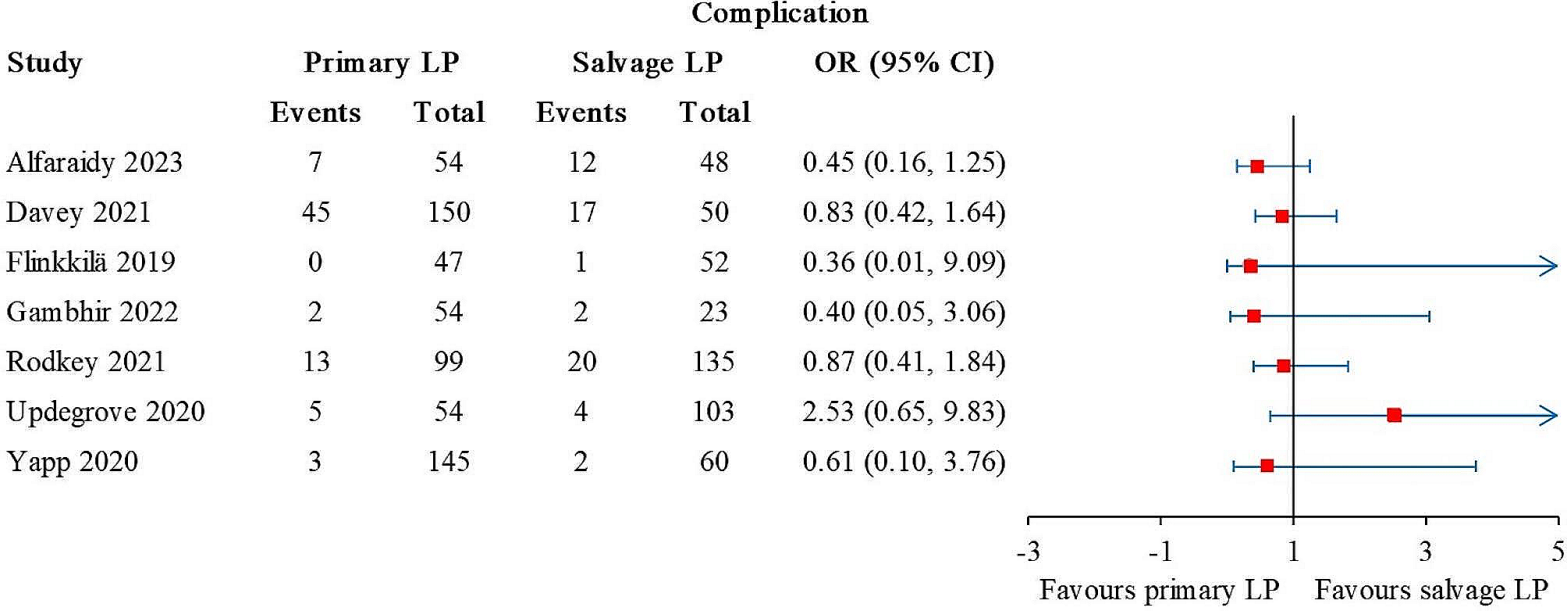
Forest plot for complications. The arrows represent values exceeding the axis scale. (CI, confidence interval; LP, Latarjet procedure.)

The complications were reported in 9 studies, of which 2 studies reported 8 and 13 complications, respectively, but did not specify how many were in the primary and salvage LP groups [27, 28]. The other 7 studies involved 603 primary LP and 471 salvage LP [21, 22, 25, 26, 35–37]. No statistical difference was detected between the patient groups (Fig. 3). The overall I^2^ value was 0%.

### Reoperations

**Fig. 4:**
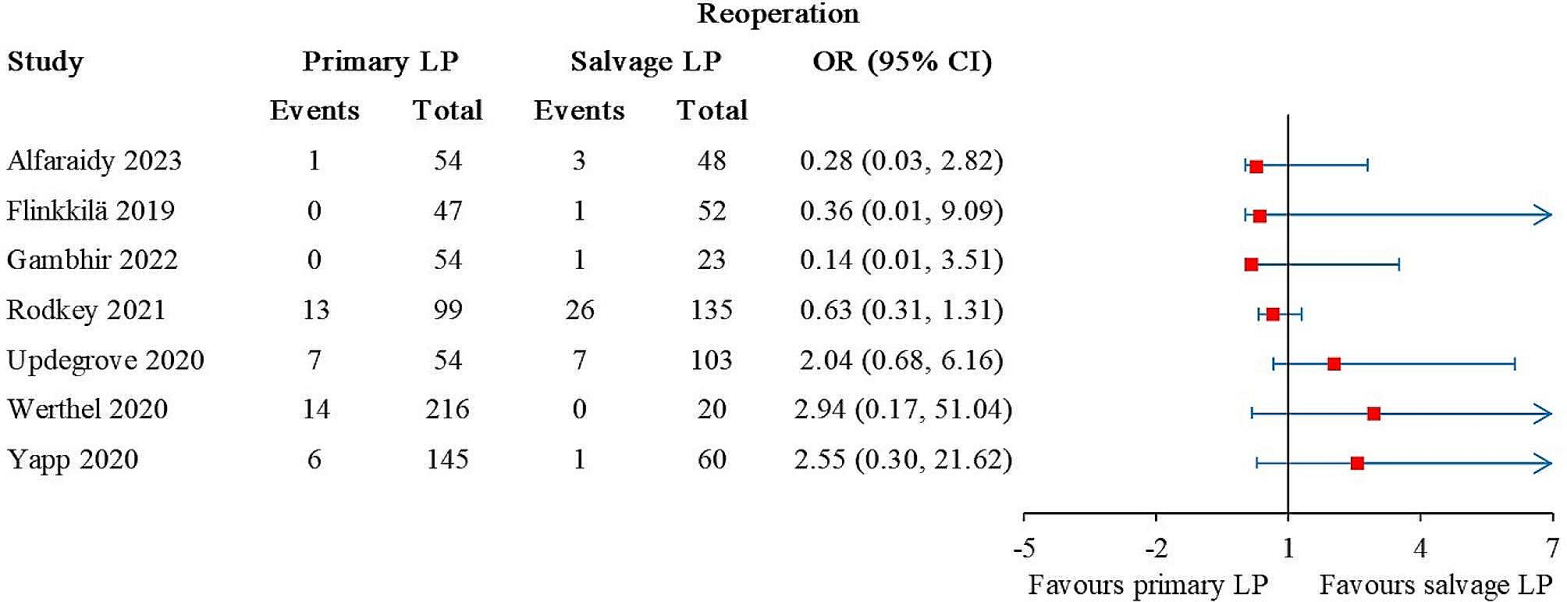
Forest plot for reoperations. The arrows represent values exceeding the axis scale. (CI, confidence interval; LP, Latarjet procedure.)

Reoperations were reported in 9 studies, of which 2 studies reported 1 and 3 reoperations, respectively, but did not specify how many were in the primary and salvage LP groups [27, 28]. The other 7 studies involved 669 primary LP and 441 salvage LP [21, 22, 26, 29, 35–37]. No statistical difference was detected between the patient groups (Fig. 4). The overall I^2^ value was 16%.

### Return to sports

**Fig. 5:**
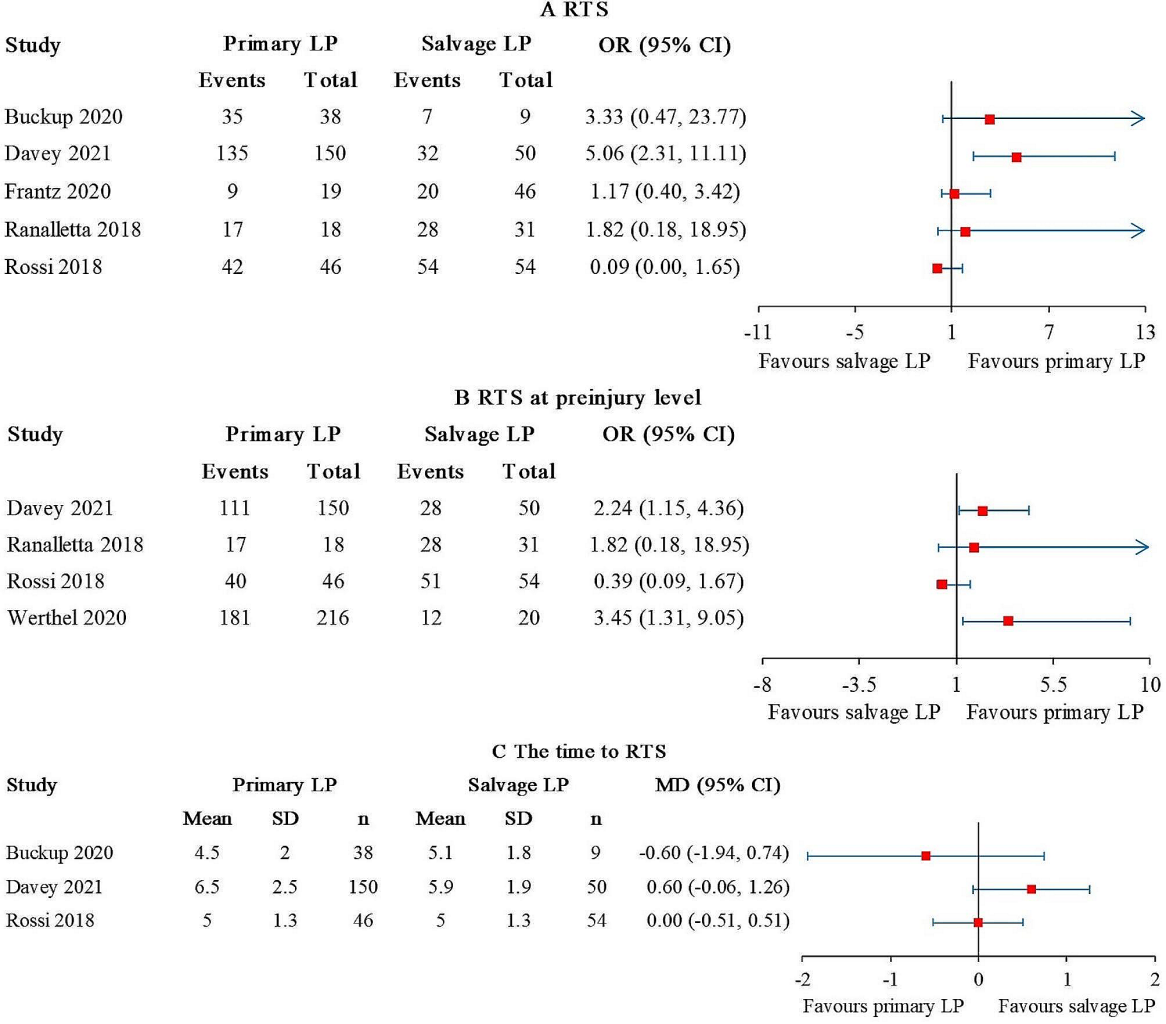
Forest plot for (**A**) RTS, (**B**) RTS at preinjury level, and (**C**) the time to RTS. The arrows represent values exceeding the axis scale. (RTS, return to the same sport; CI, confidence interval; LP, Latarjet procedure.)

The rate of returning to the same sport (RTS) was reported in 5 studies [24, 25, 27, 28, 34], comprising 271 primary LP and 190 salvage LP. A statistically significant difference was found in 1 out of 5 studies [25], and the result favored primary LP (Fig. 5A). The overall I^2^ value was 63%. When the studies conducted by Buckup et al. [24] and Ranalletta et al. [27] were excluded, the I^2^ value decreased to 0%.

The rate of RTS at preinjury level was reported in 4 studies [25, 27–29], comprising 430 primary LP and 155 salvage LP. Statistically significant differences favored primary LP were found in 2 out of the 4 studies [25, 29] (Fig. 5B). The overall I^2^ value was 52%. After excluding the study conducted by Rossi et al. [28], the I^2^ value dropped to 0%.

The time to RTS was reported in 3 studies [24, 25, 28], comprising 234 primary LP and 113 salvage LP. No statistical difference was detected between the patient groups (Fig. 5C). The overall I^2^ value was 40%.

### Patient-reported outcomes

**Fig. 6:**
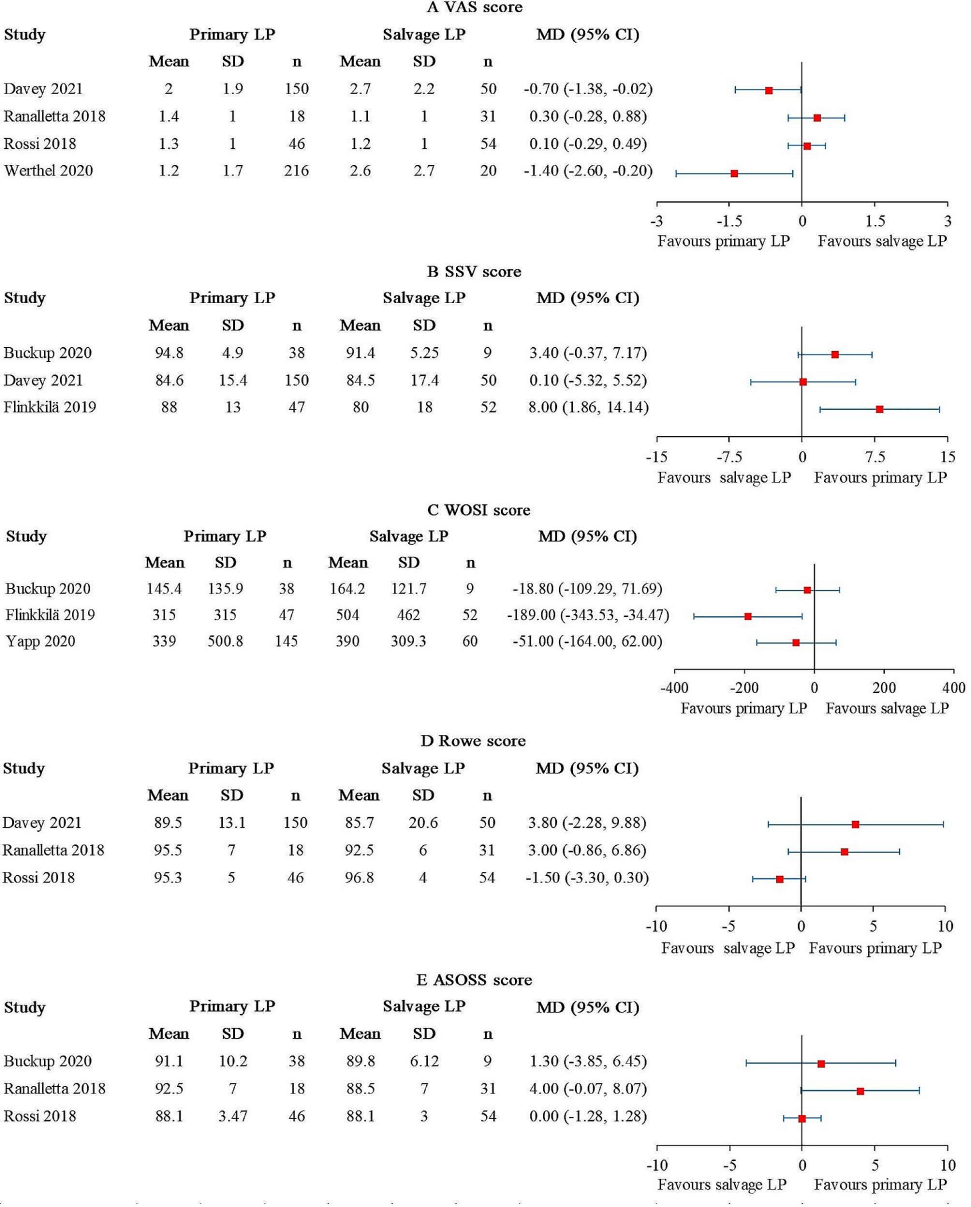
Forest plot for (**A**) VAS score, (**B**) SSV score, (**C**) WOSI score, (**D**) Rowe score, and (**E**) ASOSS score. (CI, confidence interval; LP, Latarjet procedure.)

The VAS score was reported in 4 studies [25, 27–29], comprising 430 primary LP and 155 salvage LP. Statistically significant differences favored primary LP were found in 2 out of the 4 studies [25, 29] (Fig. 6A). However, neither of the MD reached the MCID. The overall I^2^ value was 71%. When the studies conducted by Ranalletta et al. [27] and Rossi et al. [28] were excluded, the I^2^ value decreased to 0%.

The SSV score was reported in 3 studies [22, 24, 25], comprising 235 primary LP and 111 salvage LP. A statistically significant difference favored primary LP was found in 1 out of the 3 studies [22] (Fig. 6B). The MD did not reach the MCID. The overall I^2^ value was 44%.

The WOSI score was reported in 3 studies [22, 24, 26], comprising 230 primary LP and 121 salvage LP. A statistically significant difference was found in 1 out of 3 studies [22], and the result favored primary LP (Fig. 6C). The MD did not reach the MCID. The overall I^2^ value was 43%.

The Rowe score was reported in 3 studies [25, 27, 28], comprising 214 primary LP and 135 salvage LP. No statistical difference was detected between the patient groups (Fig. 6D). The overall I^2^ value was 68%. After excluding the study conducted by Rossi et al. [28], the I^2^ value dropped to 0%.

The ASOSS score was reported in 3 studies [24, 27, 28], comprising 102 primary LP and 94 salvage LP. No statistical difference was detected between the patient groups (Fig. 6E). The overall I^2^ value was 43%.

### Range of motion

**Fig. 7:**
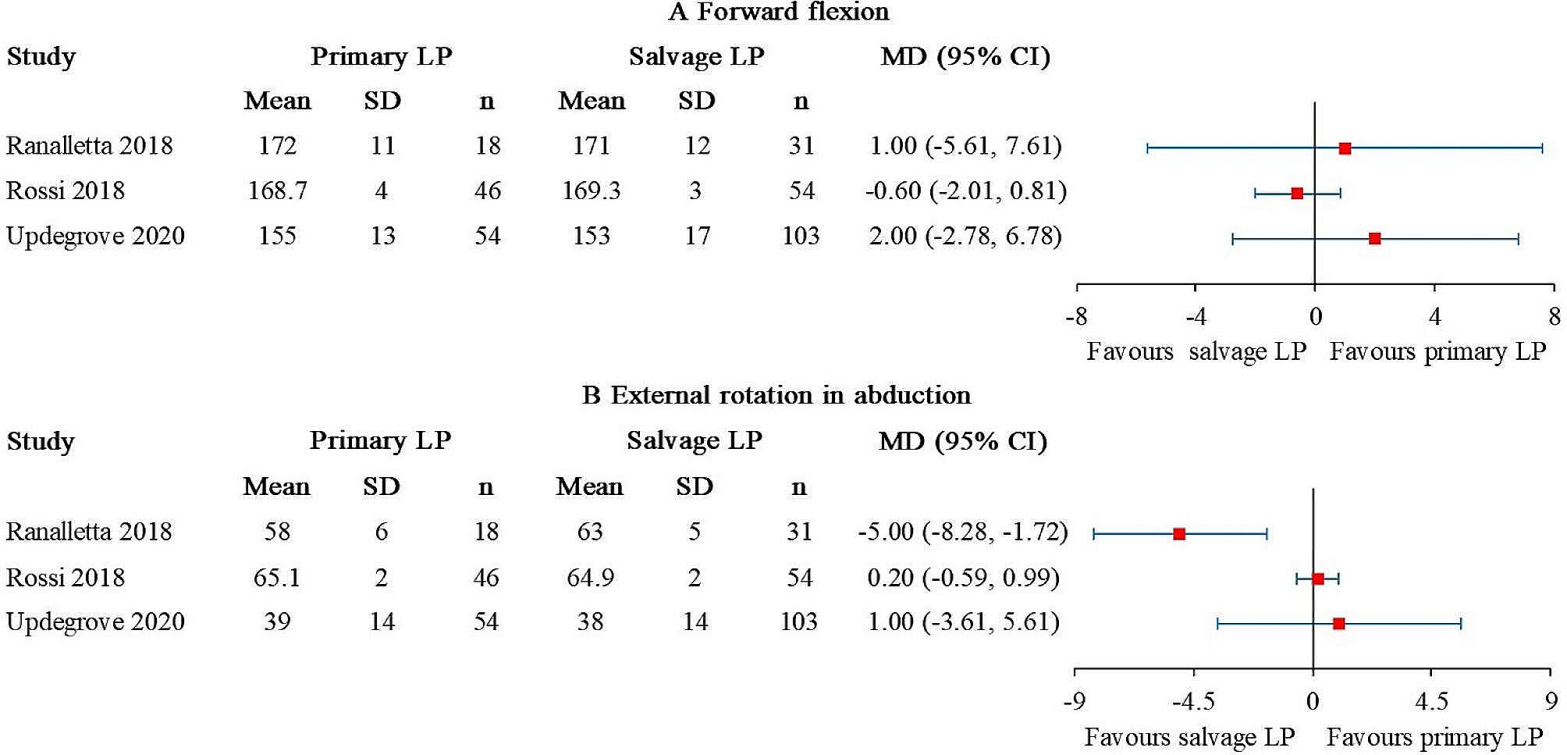
Forest plot for (**A**) forward flexion and (**B**) external rotation in abduction. (CI, confidence interval; LP, Latarjet procedure.)

Forward flexion and external rotation in abduction were reported in 3 studies [27, 28, 35], comprising 118 primary LP and 188 salvage LP (Fig. 7). No statistical difference of forward flexion was detected between the patient groups (Fig. 7A). The I^2^ value was 0%. A statistically significant difference of external rotation in abduction was found in 1 out of 3 studies, and the result favored salvage LP (Fig. 7B). The I^2^ value was 79%. After excluding the study conducted by Ranalletta et al., the I^2^ value dropped to 0%.

## Discussion

The main findings of this study were that compared with primary LP, salvage LP was likely to result in higher rate of recurrent instability and lower rate of RTS at preinjury level. Primary and salvage LP appeared to yield similar outcomes regarding complications, reoperations, the rate of RTS, the time to RTS, pain, shoulder function, and range of motion. The results of this study indicated that compared with primary LP, salvage LP might have higher risks of recurrent instability and lower rate of RTS at preinjury level, which might be related to the more critical glenoid bone loss in salvage LP group [22, 25, 28, 38]. Furthermore, the quality of anteroinferior capsule in salvage LP group was often worse than that in primary LP group [28]. Previous biomechanical cadaveric studies showed that capsular repair could enhance the effect of LP on restoring shoulder stability, whether capsule was repaired to coracoacromial ligament or glenoid rim [39, 40]. Since the capsule is one of the stabilization structures of shoulders, the poor-quality capsule in salvage LP group might be associated with the higher incidence of instability and inferior level of sports.

This study provided theoretical support for those who advocate LP as a primary stabilization procedure. Especially for high-risk shoulder instability patients and physically active individuals, LP can be performed as primary stabilization procedure to avoid reoperations due to the failure of the primary Bankart repair. Because the postoperative shoulder stability and sports level following salvage LP might be inferior to that following primary LP. Furthermore, numerous studies had also confirmed that primary LP can achieve excellent clinical outcomes for high-risk shoulder instability patients and physically active people [13, 41]. Surgeons should conduct a comprehensive preoperative assessment of shoulder instability risks and perform the primary stabilization procedure on individual basis, thereby maximizing the potential benefits for patients.

Interestingly, this study found that the primary and salvage LP appeared to have comparable efficacy in terms of complications, reoperations, the rate of RTS, the time to RTS, pain, shoulder function, and ROM. This result suggested that it might also be reasonable for some surgeons to recommend Bankart repair as a primary stabilization procedure and LP as a salvage procedure after failed prior Bankart repair for patients with glenoid bone loss less than 15–20% or lower sports demand. Because numerous studies have reported that the incidence of complications following Bankart repair was significantly lower than that following LP [42, 43], and salvage LP would not increase the risk of complications and reoperations, nor reduce the rate of RTS, shoulder function, and ROM compared with primary LP. But patients should be informed the failure of the primary stabilization procedure has a potential negative impact on the efficacy of salvage LP.

This study has the following limitations. First, the majority of studies included in this systematic review were retrospective in nature and exhibited relatively low quality of evidence, thereby diminishing the robustness of the conclusions drawn. Additionally, due to the absence of meta-analysis, it was not possible to provide a pooled effect estimate for different timing of surgery. More high-quality and large-sample comparative studies were needed to further verify the findings of this study in the future. Second, due to the limitations of the studies included in this systematic review, we were unable to analyze the effect of the type and number of prior failed stabilization procedures on outcomes in the salvage LP group. Third, although the results of this study suggested that salvage LP might result in higher rate of recurrent instability and lower rate of RTS at preinjury level compared with primary LP, we could not determine the influence of other factors (e.g., patient characteristics, surgery techniques, and rehabilitation protocols et, al.) on our findings.

## Conclusion

Current evidence suggests that compared with primary LP, the salvage LP may provide inferior postoperative outcomes in terms of recurrent instability and the rate of RTS at preinjury level. Primary and salvage LP may yield comparable efficacy in terms of complications, reoperations, the rate of RTS, the time to RTS, pain, shoulder function, and ROM.

### Electronic supplementary material

Below is the link to the electronic supplementary material.

Supplementary Material 1

Supplementary Material 2

## Data Availability

The datasets used and analysed during the current study are available from the corresponding author on reasonable request.

## Abbreviations

LP: Latarjet procedure
PRISMA: Preferred Reporting Items for Systematic Reviews and Meta-Analyses
RTS: return to the same sports
VAS: visual analog scale
SSV: subjective shoulder value
WOSI: Western Ontario Shoulder Instability Index
ASOSS: Athletic Shoulder Outcome Scoring System
PROSPERO: International prospective register of systematic reviews
MINORS: Methodological Index for Nonrandomized Studies
LOE: level of evidence
RCS: retrospective cohort study
PCS: prospective cohort study
