# Supplementary material for: Salvage Latarjet may provide worse outcomes in terms of recurrent instability and returning to sports compared to primary Latarjet: a systematic review of comparative studies
Source: BMC Musculoskelet Disord. 2024 Jun 27;25:500. doi: 10.1186/s12891-024-07593-w (PMC11212418; doi:10.1186/s12891-024-07593-w)
Supplement: Supplementary file 2 — Supplementary Material 2 [file 12891_2024_7593_MOESM2_ESM.docx]

Supplementary table 1 The evaluation of methodological quality of the included studies using MINORS.

| First Author, Year | Study Design, LOE | MINORS | | | | | | | | | | | | |
| --- | --- | --- | --- | --- | --- | --- | --- | --- | --- | --- | --- | --- | --- | --- |
|  |  | 1 | 2 | 3 | 4 | 5 | 6 | 7 | 8 | 9 | 10 | 11 | 12 | Total |
| Ranalletta, 2018^[7]^ | RCS, 3 | 2 | 2 | 2 | 2 | 2 | 2 | 2 | 0 | 2 | 2 | 0 | 2 | 20 |
| Rossi, 2018^[9]^ | RCS, 3 | 2 | 2 | 2 | 2 | 2 | 2 | 2 | 0 | 2 | 2 | 2 | 2 | 22 |
| Flinkkilä, 2019^[4]^ | RCS, 3 | 2 | 2 | 2 | 2 | 2 | 2 | 1 | 0 | 2 | 2 | 2 | 2 | 21 |
| Buckup, 2020^[2]^ | RCS, 3 | 2 | 2 | 2 | 2 | 1 | 2 | 2 | 0 | 2 | 2 | 0 | 2 | 19 |
| Frantz, 2020^[5]^ | PCS, 2 | 2 | 2 | 2 | 2 | 1 | 2 | 2 | 2 | 2 | 2 | 1 | 2 | 22 |
| Updegrove, 2020^[10]^ | RCS, 3 | 2 | 2 | 2 | 2 | 1 | 2 | 2 | 0 | 2 | 2 | 2 | 2 | 21 |
| Werthel, 2020^[11]^ | RCS, 3 | 2 | 2 | 2 | 2 | 1 | 2 | 1 | 0 | 2 | 2 | 2 | 2 | 20 |
| Yapp, 2020^[12]^ | RCS, 3 | 2 | 2 | 2 | 2 | 1 | 2 | 1 | 0 | 2 | 2 | 2 | 2 | 20 |
| Davey, 2021^[3]^ | RCS, 3 | 2 | 2 | 2 | 2 | 1 | 2 | 2 | 2 | 2 | 2 | 2 | 2 | 23 |
| Rodkey, 2021^[8]^ | RCS, 3 | 2 | 2 | 2 | 2 | 1 | 2 | 2 | 0 | 2 | 2 | 2 | 2 | 21 |
| Gambhir, 2022^[6]^ | RCS, 3 | 2 | 2 | 2 | 2 | 1 | 2 | 2 | 2 | 2 | 2 | 2 | 2 | 23 |
| Alfaraidy, 2023^[1]^ | RCS, 3 | 2 | 2 | 2 | 2 | 1 | 2 | 1 | 0 | 2 | 2 | 0 | 2 | 18 |

LOE, level of evidence; RCS, retrospective cohort study; PCS, prospective cohort study; MINORS, Methodological Index for Nonrandomized Studies. 0 points when not reported, 1 when reported but not adequate, and 2 when reported and adequate. The ideal maximum scores for noncomparative studies and comparative studies are 16 points and 24 points, respectively. Criteria: (1) a clearly stated aim, (2) inclusion of consecutive patients, (3) prospective collection of data, (4) end points appropriate to the aim of the study, (5) unbiased assessment of the study end point, (6) follow-up period appropriate to the aim of the study, (7) loss to follow-up less than 5%, (8) prospective calculation of the study size, (9) an adequate control group, (10) contemporary groups, (11) baseline equivalence of groups, and (12) adequate statistical analyses.

[1] Alfaraidy M, Alraiyes T, Moatshe G, et al. Low rates of serious complications after open Latarjet procedure at short-term follow-up[J]. J Shoulder Elbow Surg, 2023,32(1):41-9. Doi: 10.1016/j.jse.2022.06.004.

[2] Buckup J, Sternberg C, Smolen D, et al. Functional outcome and return to sports after the arthroscopic latarjet procedure in young and physically active patients[J]. Arch Orthop Trauma Surg, 2020,140(10):1487-94. Doi: 10.1007/s00402-020-03513-4.

[3] Davey MS, Hurley ET, O'Doherty R, et al. Open Latarjet Procedure in Athletes Following Failed Prior Instability Surgery Results in Lower Rates of Return to Play[J]. Arthroscopy, 2021,37(8):2412-7. Doi: 10.1016/j.arthro.2021.03.062.

[4] Flinkkilä T, Knape R, Nevalainen M, et al. Previous arthroscopic Bankart repair is an independent risk factor for an inferior outcome after Latarjet procedure[J]. Orthop Traumatol Surg Res, 2019,105(8):1481-5. Doi: 10.1016/j.otsr.2019.06.020.

[5] Frantz TL, Everhart JS, Cvetanovich GL, et al. Are Patients Who Undergo the Latarjet Procedure Ready to Return to Play at 6 Months? A Multicenter Orthopaedic Outcomes Network (MOON) Shoulder Group Cohort Study[J]. Am J Sports Med, 2020,48(4):923-30. Doi: 10.1177/0363546520901538.

[6] Gambhir N, Alben MG, Kim MT, et al. No Differences in 90-Day Complications and Admissions After Latarjet Procedure for Primary Bone Loss Versus Latarjet Procedure for Failed Arthroscopic Instability Repair[J]. Arthrosc Sports Med Rehabil, 2022,4(5):e1647-e51. Doi: 10.1016/j.asmr.2022.06.010.

[7] Ranalletta M, Rossi LA, Bertona A, et al. Modified Latarjet Without Capsulolabral Repair in Rugby Players With Recurrent Anterior Glenohumeral Instability and Significant Glenoid Bone Loss[J]. Am J Sports Med, 2018,46(4):795-800. Doi: 10.1177/0363546517749586.

[8] Rodkey DL, Colantonio DF, LeClere LE, et al. Latarjet After Failed Arthroscopic Bankart Repair Results in Twice the Rate of Recurrent Instability Compared With Primary Latarjet[J]. Arthroscopy, 2021,37(11):3248-52. Doi: 10.1016/j.arthro.2021.04.059.

[9] Rossi LA, Bertona A, Tanoira I, et al. Comparison Between Modified Latarjet Performed as a Primary or Revision Procedure in Competitive Athletes: A Comparative Study of 100 Patients With a Minimum 2-Year Follow-up[J]. Orthop J Sports Med, 2018,6(12):2325967118817233. Doi: 10.1177/2325967118817233.

[10] Updegrove GF, Buckley PS, Cox RM, et al. Latarjet Procedure for Anterior Glenohumeral Instability: Early Postsurgical Complications for Primary Coracoid Transfer Versus Revision Coracoid Transfer After Failed Prior Stabilization[J]. Orthop J Sports Med, 2020,8(6):2325967120924628. Doi: 10.1177/2325967120924628.

[11] Werthel JD, Sabatier V, Schoch B, et al. Outcomes of the Latarjet Procedure for the Treatment of Chronic Anterior Shoulder Instability: Patients With Prior Arthroscopic Bankart Repair Versus Primary Cases[J]. Am J Sports Med, 2020,48(1):27-32. Doi: 10.1177/0363546519888909.

[12] Yapp LZ, Nicholson JA, McCallum C, et al. Latarjet as a primary and revision procedure for anterior shoulder instability - A comparative study of survivorship, complications and functional outcomes in the medium to long-term[J]. Shoulder Elbow, 2020,12(5):338-48. Doi: 10.1177/1758573219864926.
